# Differential expression of nitric oxide synthases in porcine aortic endothelial cells during LPS-induced apoptosis

**DOI:** 10.1186/1476-9255-9-47

**Published:** 2012-11-26

**Authors:** Chiara Bernardini, Francesca Greco, Augusta Zannoni, Maria Laura Bacci, Eraldo Seren, Monica Forni

**Affiliations:** 1 Department of Veterinary Medical Sciences-DIMEVET, University of Bologna, Via Tolara di Sopra 50, 40064, Bologna, Ozzano dell’Emilia, Italy

**Keywords:** Endothelial cells, Endotoxin, Apoptosis, NO

## Abstract

**Background:**

It is well known that nitric oxide (NO) is generated by a family of constitutively (nNOS and eNOS) or inducibly (iNOS) expressed enzymes and takes part in different aspects of the inflammatory response; nevertheless, its effective role in the pathogenesis of multiple organ dysfunction and septic shock is not fully understood.

**Methods:**

To investigate the Nitric Oxide Synthases (NOSs) expression in endothelial cells during endotoxin exposure and the involvement of NO in lipopolysaccharide (LPS)-induced apoptosis, primary cultures of porcine Aortic Endothelial Cells (pAECs) were exposed to LPS for different time periods (1-24 h) and to LPS + L-NAME (15 h).

**Results:**

Lipopolysaccharide induced an increase in mRNA and protein iNOS expression; on the contrary, the expression of eNOS was decreased. Furthermore, NOSs localisation was in part modified by LPS treatment. No alteration in the total level of Nitric Oxide was observed. L-NAME (5 mM) addition determined a slight decrease of LPS-induced apoptosis.

**Conclusions:**

Endotoxin treatment strongly influenced NOS expression with an upregulation of iNOS and a simultaneous down regulation of eNOS. Moreover, in our model, the involvement of NO on LPS-induced apoptosis is very modest, suggesting that different pathways are involved in the regulation of this process.

## Introduction

Several studies have suggested a key role of the endothelium in the pathophysiology of severe sepsis; during this process, LPS (lipopolysaccharide), a component of the bacterial wall is able to cause structural and functional alterations of the endothelial phenotype and eventually endothelial cell death [1]. The loss of balance between pro-inflammatory and protective gene products is essential for converting endothelial cell activation, which represents a normal adaptive response to various stimuli, into endothelial dysfunction [2,3]. Since LPS-induced apoptosis is regulated by a complex pathway of signals, the identification of the molecules involved in this process is important. Our previous data demonstrated that LPS induces apoptosis in a model of a primary culture of porcine aortic endothelial cells (pAECs) and is effective in evoking “the heat shock response” with an increase of non-specific protective molecules, such as Hsp70 and Hsp32, and a specific growth factor, such as Vascular Endothelial Growth Factor (VEGF) [4].

It is well known that nitric oxide (NO) is generated by a family of constitutively (nNOS and eNOS) or inducibly (iNOS) expressed enzymes [5] and takes part in different aspects of the inflammatory response; nevertheless, its effective role in the pathogenesis of multiple organ dysfunction and septic shock is not fully understood [6].

The difficulty in clarifying the role of NO during septic shock is also a consequence of species-specific variability; in fact, many differences in the pathogenesis of septic shock between humans and rodents have been reported; plasma nitrite and nitrate concentrations detected in patients with hypotensive septic shock are lower in humans than in rodents [7]; moreover, human hepatocytes [8] and human macrophages [9,10] produce less NO than cells isolated from rodents. Furthermore, it has been described [10] that porcine macrophages, like human but unlike bovine and rat cells, are not able to produce NO *in vitro* suggesting that interspecies differences are also maintained in *in vitro* models.

The results obtained by the use of larger animal models [11-13] seem to indicate the pig as a better model of septic shock for the investigation of this disease in human.

Taking into account all these considerations, the aim of the present study was to investigate the ability of endotoxin exposure to influence eNOS and iNOS expression in terms of mRNA, protein amount and protein localisation in our model of LPS-induced apoptosis in pAECs [4]. The effect of nitric oxide in LPS-induced apoptosis was also evaluated.

## Materials and methods

### Cell cultures

Porcine Aortic Endothelial Cells were isolated as previously described [4]. The cells were cultured in Human Endothelial Basal Growth Medium (Gibco-Invitrogen, Paisley, UK) supplemented with 5% Foetal Bovine Serum (FBS, Gibco-Invitrogen) and 1% antibiotic/antimicotic (Gibco-Invitrogen). Cell number and viability (95%) were determined using a Thoma chamber under a phase-contrast microscope after vital staining with trypan blue dye. The cells were placed in T-25 tissue culture flasks (approximately 3x10^5^ cells/flask) (T25 Falcon Beckton-Dickinson, Franklin Lakes, NJ, USA) in a 5% CO_2_ atmosphere at 38.5°C. The cells were maintained in a logarithmic growth phase by routine passages every 2–3 days at a 1:3 split ratio.

### Cell treatments

All experiments were performed with cells from the third to the eighth passage.

Previous observations indicated that the time of culture could influence gene expression in a primary culture of pAECs; therefore, we decided to utilise a relative time control point for each time of treatment. The pAECs were grown until confluence in a flat bottom 24-well assay plate (approximately 4x10^4^ cells/well) (353813 Falcon Beckton-Dickinson) or, for immunohistochemical study, in 8-well slide chambers (approximately 4x10^4^ cells/well) (354631 Beckton-Dickinson).

Lipopolysaccharide 10 μg/ml (*E. coli* 055:B5, Sigma-Aldrich Co, St Louis, MO, USA) was added to the culture medium for different time periods (1, 2, 3, 4, 5, 6, 7, 15 and 24 h for iNOS and eNOS mRNA expression study; 7, 15, 24 h for iNOS and eNOS protein expression and immunolocalisation; 1, 3, 7, 15 and 24 h for NO level determination. Control samples were utilised for each time period of treatment. At the end of each experimental point, treated or control cells and media were collected and stored until the analysis.

L-NAME 5 mM or 10 mM (N5751, Sigma) was added to the culture with or without LPS (10 μg/ml) and incubated for 15 h. At the end of treatment the total levels of NO and the apoptosis rate were evaluated.

### Real-time PCR quantification of iNOS and eNOS mRNA

Total ribonucleic acid (RNA) from pAECs was isolated using an RNeasy Mini Kit 50 (Qiagen Sciences Inc, MD, USA) and treated with RNase-free DNase kit (Qiagen) according to the manufacturer’s instructions.

The RNA concentration was spectrophotometrically quantified (A_260_ nm) and 1 μg of total RNA was reverse transcribed to cDNA using an iScript cDNA Synthesis Kit (Bio-RAD Laboratories Inc., California, USA) at a final volume of 20 μl. Swine primers (eNOS, iNOS and Hypoxanthine-guanine phosphoribosiyltransferase -HPRT) were designed using Beacon Designer 2.07 Software (premier Biosoft International, Palo Alto, Ca, USA). Their sequences, expected PCR product length and accession number in the EMBL database are shown in Table 1. Real-time quantitative polymerase chain reaction (PCR) was carried out in an iCycler Thermal Cycler (Bio-RAD) using SYBR green I detection. A master-mix of the following reaction components was prepared to the indicate end-concentrations: forward primer (0.2 μM) and reverse primer (0.2 μM), 1x IQ SYBR Green BioRad Supermix (Bio-RAD Laboratories Inc.). Then, 2.5 μl of cDNA was added to 22.5 μl of the master mix. All samples were carried out in duplicate. The following real-time PCR protocol was employed: initial denaturation for 3 min at 95°C, 40 cycles of 95°C for 15 sec and 60°C for 30 sec, followed by a melting step with slow heating from 55°C-95°C with a rate of 0.5°C/s. The housekeeping gene HPRT data were used to normalise the amount of RNA.

**Table 1:** Forward and reverse primer sequences, RT-PCR product length and accession number (Acc.No.) in the EMBL database

**Primer**	**Sequence (5^′^-3^′^)**	**Product Length (bp)**	**Acc. No.**
eNOS	For.: GCTCTCACCTTCTTCCTG	144	AY266137
	Rev.: CCACTTCCACTCCTCATAG		
iNOS	For.: CAACAATGGCAACATCAGG	119	U59390
	Rev.: CATCAGGCATCTGGTAGC		
HPRT	For.: GGACAGGACTGAACGGCTTG	115	AF143818
	Rev.: GTAATCCAGCAGGTCAGCAAAG		

The expression of iNOS and eNOS mRNA under standard culture conditions was calculated as a threshold cycle (deltaC_T_) (HPRT C_T_ – iNOS/eNOS C_T_). The expression of iNOS and eNOS mRNA during LPS treatment was calculated as the fold of increase using the ΔΔCt method (ABI Prism 7700 Sequence detection System User Bulletin #2. Relative quantification of gene expression; 1997 and 2001).

Real-time efficiency for each primer set was acquired by amplification of a standardised cDNA dilution series. The specificity of the amplified PCR products was verified by analysis of the melting curve, which is sequence-specific and by an agarose gel electrophoresis run.

### iNOS and eNOS Western blot

The cells were harvested and lysed in SDS solution (Tris–HCl 50 mM pH 6.8; SDS 2%; glycerol 5%). The protein content of cellular lysates was determined by a Protein Assay Kit (TP0300, Sigma). Aliquots containing 20 μg of proteins were separated on NuPage 4-12% bis-Tris Gel (Gibco-Invitrogen) for 50 min at 200 V. The proteins were then electrophoretically transferred onto a nitrocellulose membrane. The blots were washed in PBS and protein transfer was checked by staining the nitrocellulose membranes with 0.2% Ponceau Red. Non-specific binding on nitrocellulose membranes was blocked with 5% milk powder in PBS-T20 (Phosphate Buffer saline-0.1% Tween-20) for 1 h at room temperature. The membranes were then incubated overnight at 4°C with a 1:200 dilution of an anti-iNOS (610332, BD Transduction) rabbit polyclonal antibody in PBS-T20 with 3% milk powder or at 4°C overnight with a 1:500 dilution of an anti-eNOS mouse monoclonal antibody (Sa-258, Biomol, Butler Pike, Plymouth Meeting, PA, USA) in Tris Buffered Saline-T20 (TBS-T20 20 mM Tris–HCl, pH 7.4, 500 mM NaCl, 0.1% T-20). After several washings with PBS-T20, the membranes were incubated with the secondary biotin-conjugate antibody and then with a 1:1000 dilution of an anti-biotin horseradish peroxidase (HRP)-linked antibody. The western blots were developed using chemiluminescent substrate (Super Signal West Pico Chemiluminescent Substrate, Pierce Biotechnology, Inc, Rockford, IL, USA) according to the manufacturer’s instructions. The intensity of the luminescent signal of the resultant bands was acquired by Fluor-S^TM^ Multimager using Quantity One Software (Bio-RAD).

In order to normalise the NOS data on the housekeeping protein, membranes were stripped (briefly: the membranes were washed 5 min in water, then 5 min in 0.2 M NaOH and then washed again in water) and re-probed for housekeeping β-tubulin (1:500 sc-5274 Santa Cruz Biotechnology Inc, Santa Cruz, CA, USA).

The relative protein content (NOS/ β-tubulin) was expressed as arbitrary units (AUs).

### iNOS and eNOS protein localisation by immunofluorescence staining

The cells were fixed with ethanol:acetic acid (2:1) for 10 min at −20°C, and permeabilised with 0.1% Triton X-100. Thereafter, the cells were washed with PBS three times and incubated for 1 h at RT with 10% FBS in DPBS (Dulbecco Phosphate Buffer Solution, Cambrex Bio-Science INC, Wakersville, MA, USA). Primary antibodies: 1:100 anti-iNOS (BD Transduction) and 1:100 anti eNOS (Biomol) were added, and the slides were incubated in a humidified chamber at 4°C overnight. After several washes with PBS, a 1:800 dilution of fluorescein isothiocyanate (FITC)-conjugated secondary antibodies was added. The cells were then counterstained with propidium iodide (PI) and examined under an epifluorescence microscope (Eclipse e600 Nikon, Japan) equipped with appropriate filters and a Nikon DXM 1200 digital camera with ACT-2U software for DMX 1200.

### NO determination

The NO concentrations in culture media were determined by the “Total Nitric Oxide Assay” (DE 1600 R&D Systems, Minneapolis, MN 55413, USA) using Appliskan 100-240 V (Thermo Electron Corporation, Vantaa Finland). This assay determines total nitric oxide concentration based on the enzymatic conversion of nitrate to nitrites by nitrate reductase. The reaction is followed by a colorimetric detection of nitrite as an azo dye product of the Griess Reaction. The sensitivity of the assay was less than 1.35 μmol/L, and the intra- and interassay coefficients of variation were less than 5% and 7% respectively. The basal level of NO present in the fresh endothelial medium was subtracted from all samples (CTR or treated cells).

### Apoptosis detection

A photometric enzyme immunoassay for the qualitative and quantitative in vitro determination of cytoplasmic histone-associated DNA fragments mono- and oligonucleosomes was carried out according to the manufacturer’s instructions (1774425, Roche Diagnostics GmbH, 82372 Penzberg, Germany) using Appliskan 100-240 V (Thermo Electron Corporation, Vantaa Finland). Briefly, the cells were lysed directly in the well, and the cytoplasmatic and nuclear fractions were separated by centrifugation at 200 x *g*. Twenty microliters of cytoplasmatic fraction were added to a streptavidin-coated microtiter plate. The biotin-labeled anti-histone antibody was added, followed by an HRP-conjugated anti-DNA antibody. The reaction of the nucleosome-bound DNA fragments was quantified using photometric analysis.

### Statistical analysis

Each treatment was replicated three times (mRNA and protein analysis) or six times (NO and apoptosis detection) in three independent experiments (n = 3). The data were analysed by a one-way analysis of variance (ANOVA) followed by the Tukey *post hoc* comparison Test (SPSS program version 13.0; SPSS Inc, Chicago, IL, USA). Differences of at least p < 0.05 were considered significant.

## Results

Standard culture conditions did not affect NOS expression or NO production.

**Figure 1:**
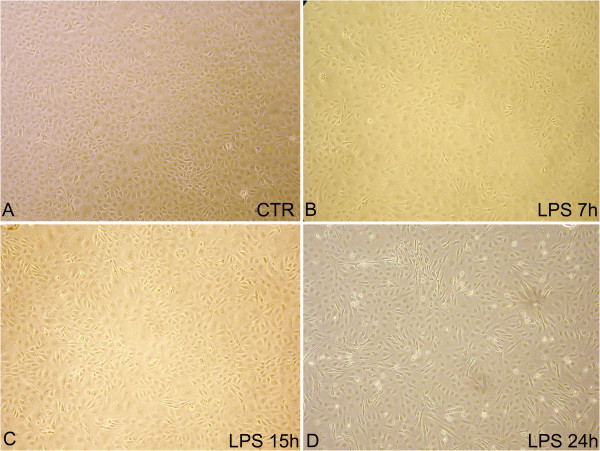
**Representative images of pAEC morphology.A**) pAECs under standard culture conditions. **B**, **C**, **D**) pAECs after 7, 15 and 24 h of LPS treatment, respectively (phase contrast microscope 200X). LPS treatment induced a moderate spindle-shape morphological change well evidenced after 24 h.

The morphology of an untreated pAEC primary culture is shown in Figure 1A. Lipopolysaccharide treatment induced a moderate spindle-shaped morphological change which was well evidenced after 24 h (Figure 1B-D).

### Quantification of iNOS and eNOS mRNA expression

**Figure 2:**
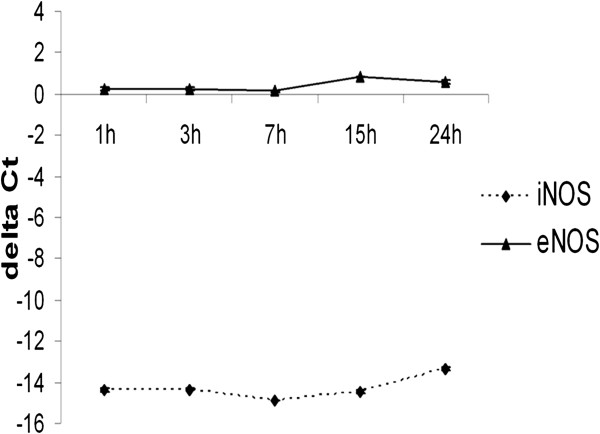
**Relative gene expression of iNOS and eNOS in pAECs under standard culture conditions at different time periods.** Relative mRNA data are expressed as delta Ct (HPRT Ct - iNOS or eNOS Ct). The data represent the mean ± SEM (n = 3). Time of culture did not influence iNOS and eNOS mRNA expression.

Under standard culture conditions, the iNOS mRNA expression level was notably lower than eNOS (Figure 2).

**Figure 3:**
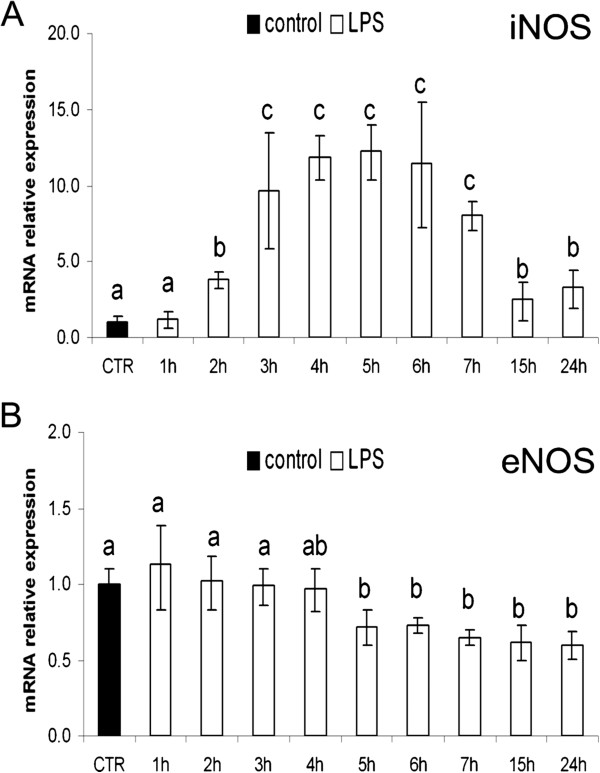
**Relative gene expression of iNOS (A) and eNOS (B) in pAECs treated with LPS (10** **μg/ml) at different time periods (n = 3).** LPS increased iNOS mRNA expression while eNOS mRNA expression was decreased. Relative mRNA data are expressed as the fold of increase (ΔΔCt method) in respect to the control (CTR = mean ± SEM of all control time points). Error bars represent the range of relative expression. The different letters above the bars indicate significant differences in the various time points (p < 0.05, ANOVA post hoc Tukey’s test).

Lipopolysaccharide induced a significant increase of iNOS mRNA levels starting after 2 h of continuous treatment, remaining stable until 7 h and declining at 15 and 24 h, without reaching the iNOS mRNA level in the pAECs cultured under standard conditions (Figure 3A).

Conversely, eNOS mRNA levels were decreased by LPS treatment starting after 5 h of continuous treatment until the end of the experiment (Figure 3B).

### Quantification of iNOS and eNOS protein expression

**Figure 4:**
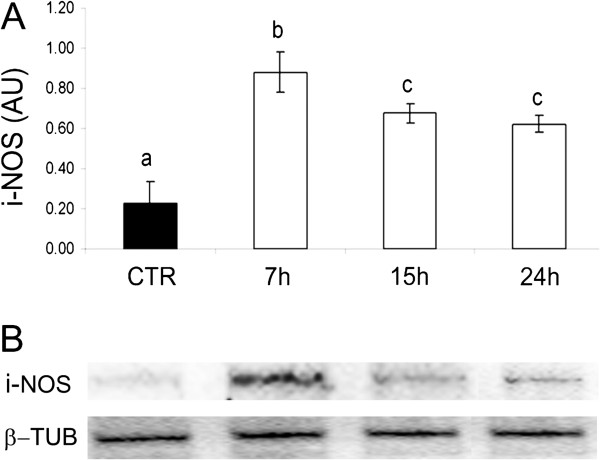
**Expression of iNOS protein in pAECs treated with LPS (10** **μg/ml) for different time periods (7, 15, 24 h).A**) LPS induced a significant increase of iNOS protein expression with respect to the control (CTR = mean ± SEM of all control time points). The data represent the mean ± SEM (n = 3) of relative protein content (AU = Arbitrary Units). The different letters above the bars indicate significant differences among the various time points (p < 0.05, ANOVA post hoc Tukey’s test, n = 3). **B**) Representative Western Blot of iNOS and relative housekeeping β-tubulin were reported.

The iNOS protein level detected in the pAECs cultured under standard culture conditions was very low while the LPS treatment induced a significant increase of iNOS at 7 h; the protein content then decreased without reaching the basal level of the control cells (Figure 4A).

**Figure 5:**
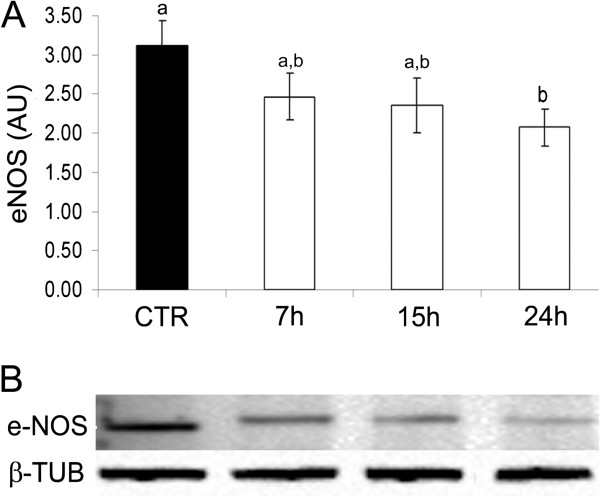
**Expression of eNOS protein in pAECs treated with LPS (10** **μg/ml) for different time periods (7, 15, 24 h).A**) LPS induced a significant decrease of eNOS protein expression in respect to the control (CTR = mean ± SEM of all control time points). The data represent the mean ± SEM (n = 3) of the relative protein content (AU = Arbitrary Units). The different letters above the bars indicate significant differences among the various time points (p < 0.05, ANOVA post hoc Tukey’s test, n = 3). **B**) Representative Western Blot of eNOS and relative housekeeping β-tubulin were reported.

The eNOS protein is largely expressed in pAECs cultured under standard conditions while LPS treatment induced a significant decrease of eNOS protein expression (Figure 5A).

### iNOS and eNOS protein localisation

**Figure 6:**
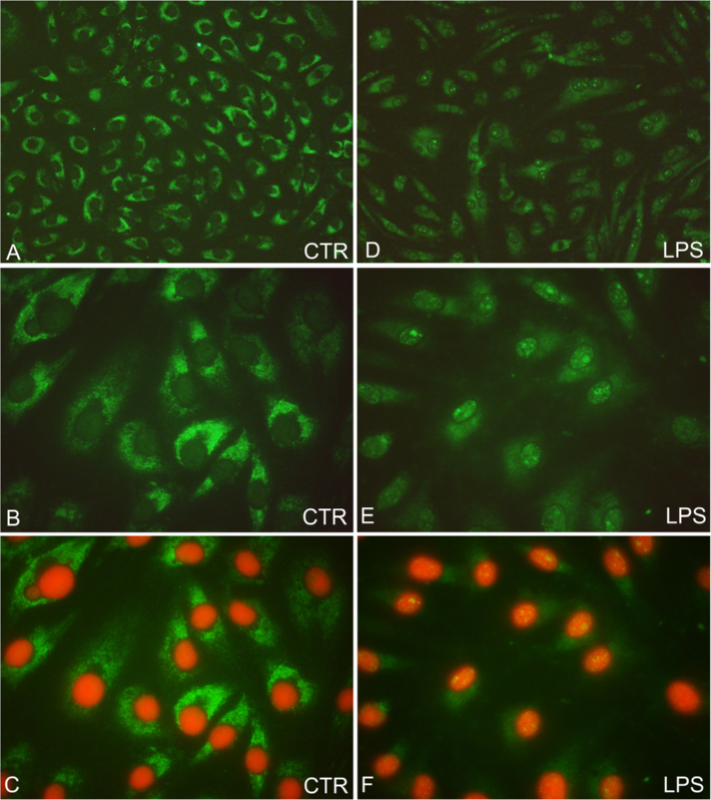
**Representative iNOS immunofluorescent staining on pAECs.** Green fluorescence indicates positive immunostaining revealed by FITC, red fluorescence indicates nuclear staining with Propidium Iodide (A, D magnification 200X; B, C, E, F magnification 1000X). **A**-**C**) iNOS protein localization in standard cultured pAECs; a cytoplasmatic signal is evidenced. **D**-**F**) iNOS protein localisation after LPS treatment (15 h); a nuclear signal in the form of bright granules is revealed.

The iNOS protein was detected in the cytoplasm of the pAECs cultured under standard culture conditions (Figure 6A-C) while, in LPS treated cells, in addition to a cytoplasmatic signal, nuclear staining was detected (Figure 6D-F).

**Figure 7:**
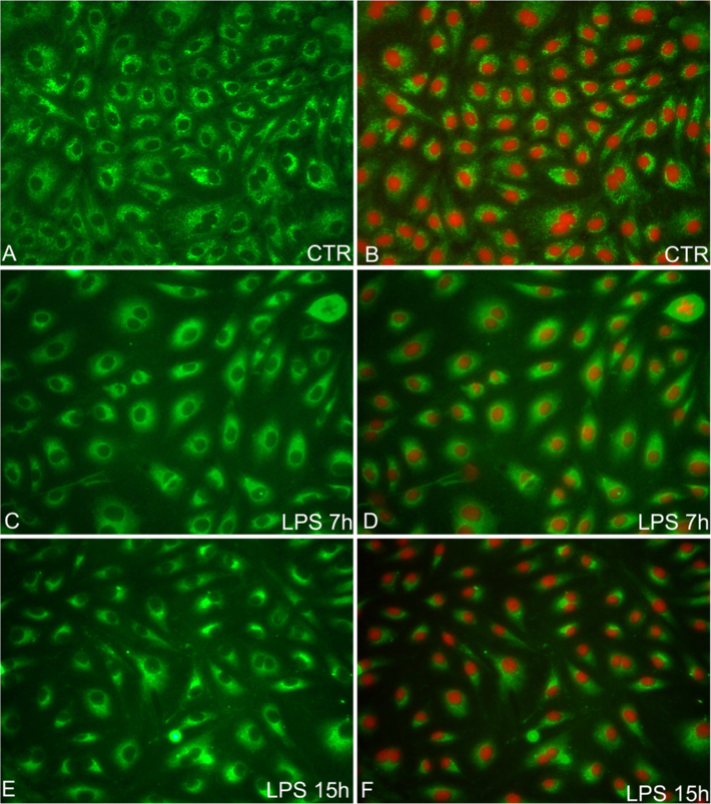
**Representative eNOS immunofluorescent staining on pAEC.** Green fluorescence indicates positive immunostaining revealed by FITC, red fluorescence indicates nuclear staining with Propidium Iodide (magnification 400X). **A**, **B**) eNOS protein localisation in standard cultured pAECs; a cytoplasmatic signal evenly diffused was detected. (**C**-**F**) LPS treatment resulted in a modification of the signal with more intense staining in the perinuclear region.

Under standard culture conditions, the eNOS protein is diffusely distributed in the cytoplasm of pAECs (Figure 7A, B); LPS treatment resulted in a modification of the signal with more intense staining in the perinuclear region (Figure 7C-F).

### Quantification of total NO level

**Figure 8:**
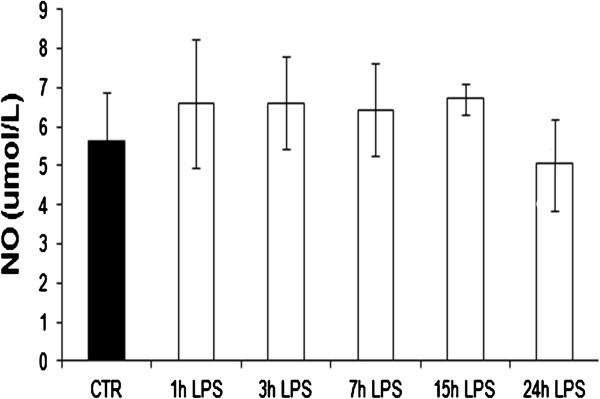
**The effect of LPS treatment on NO level (μmol/L) in the culture medium of the pAECs.** The cells are treated with LPS (10 μg/ml) (white bars) for 1, 3, 7, 15 and 24 h (CTR = mean ± SEM of all control time points, black bars). The data represent the mean ± SEM (n = 3). No significant differences are observed.

**Figure 9:**
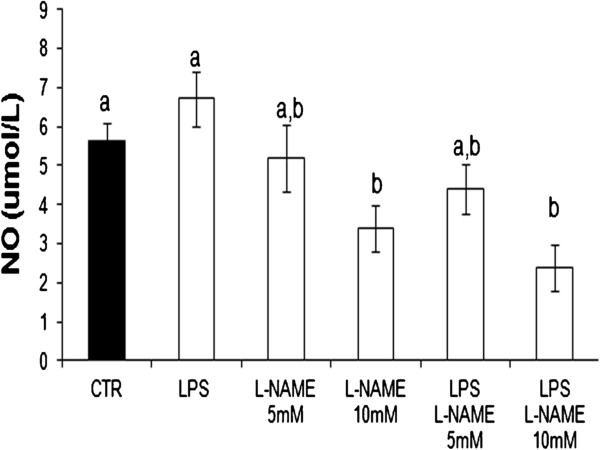
**The dffect of L-NAME on NO levels in pAEC culture medium.** The pAECs were treated with L-NAME (5 or 10 mM) in the presence or absence of LPS (10 μg/ml 15 h). The data represent the mean ± SEM (n = 3). The different letters above the bars indicate significant differences in the various time points (p < 0.05, ANOVA post hoc Tukey’s test).

Lipopolysaccharide treatment did not induce any significant change in the total level of nitric oxide (Figure 8). L-NAME (5-10 mM) induced a dose dependent decrease of total NO, both under standard control conditions and with LPS treatment (Figure 9).

### L-NAME effect on LPS-induced apoptosis

**Figure 10:**
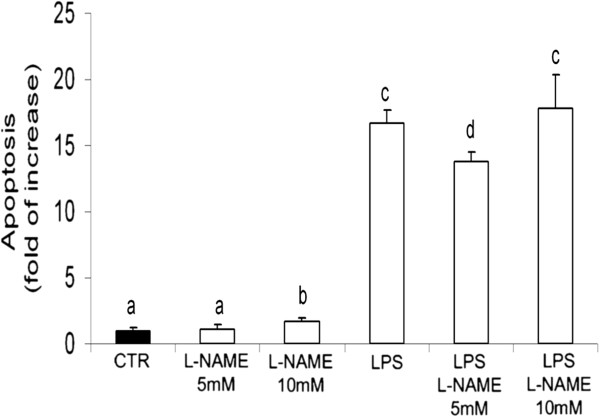
**Effects of L-NAME treatment on pAEC LPS-induced apoptosis.** Cells are treated with or without LPS (10 μg/ml) for 15 h, with or without L-NAME (5 or 10 mM). The apoptosis level was calculated as the fold of increase with respect to the control (CTR) Data represent the mean ± SEM (n = 3). The different letters above the bars indicate significant differences in the various time points (p < 0.05, ANOVA post hoc Tukey’s test).

Lipopolysaccharide induced a significant increase of the apoptosis rate in pAECs while L-NAME treatment (5 mM) partially protected cells against LPS-induced apoptosis (Figure 10). L-NAME 10 mM produced a slight but significant increase of the apoptosis rate in pAECs cultured under standard culture conditions and was unable to reduce LPS-induced apoptosis.

## Discussion

The present study demonstrated that LPS influenced the expression of eNOS and iNOS in a primary culture of pAECs.

Endotoxin treatment deeply influenced the expression of eNOS and iNOS with an opposite effect: a significant up-regulation of the inducible iNOS and a significant down-regulation of the constitutive eNOS. This effect was demonstrated by both mRNA and the protein data; in fact, eNOS mRNA is reduced after 5 h of LPS treatment, and these low levels remain constant and do not return to the basal amount. The eNOS protein level confirmed and reflected the mRNA kinetic trend. The iNOS mRNA up-regulation is more intense and constant from 3 to 7 h of LPS treatment while, after 15 and 24 h iNOS stimulation is lower even if it does not return to the basal level.

Despite the fact that the effect of LPS on the expression of NOSs is evident, the nitric oxide levels recorded in the culture medium after the administration of the endotoxin did not undergo significant changes. To explain this result, we must, first of all, consider that the determination of the total levels of NO in the endothelial culture medium is extremely difficult due to the high concentrations of NO present in this specific medium, as indicated by Papapetropoulos et al. [14]. In order to reduce the effect of the culture medium on NO detection, we subtracted the basal level of the NO present in the medium of standard culture conditions from all values. The data thus obtained, however, do not show significant variations as has also been indicated by other authors who showed no significant changes in the levels of NO following LPS administration in the same cellular line [15] but also in mouse [16] and bovine [17] endothelial cells. Therefore, our hypothesis was that the elevated presence of NO in basal culture medium may mask the small change of NO level generated by the decrease of eNOS expression and the simultaneous increase of iNOS expression.

Increasing evidence [18-20] demonstrated the different roles performed by NOS enzymes on the basis of different cellular localisations. In our model we demonstrated that endotoxin exposure influenced NOS localisation. Under standard culture conditions, iNOS is detected as a cytoplasmatic signal while after LPS treatment, the signal is evident in the nuclear region. The presence of iNOS in the nucleus was demonstrated in different cellular types including brown adipocytes [21], rat neutrophils [18] and rat VSMC [22]. The meaning of the nuclear presence of iNOS is not well clarified but the hypothesis that NO could exert a toxic effect, caused by direct damage of the DNA or indirect damage by affecting gene transcription and cell proliferation, was formulated [23]. Constitutive eNOS is diffusely distributed in the cytoplasm of pAECs in accordance with the multiple functions of eNOS in ECs physiological condition. Lipopolysaccharide treatment determined more intense staining in the perinuclear region in accordance with that observed by Iwakiri et al. [19] and Zhang et al. [20]. Additional investigation is necessary to clarify the functional significance of eNOS distribution after LPS treatment, taking into account the complex change in endothelial cell morphology induced by endotoxins [24,25].

The last step of our study was to evaluate the involvement of eNOS and iNOS activity on LPS-induced apoptosis. As indicated by other authors [14], an elevated concentration of L-NAME in the range of mM is necessary to block NO release in the culture medium of endothelial cells. Our results indicated that L-NAME 10 mM increased the apoptosis rate, thus suggesting a toxic effect of this dose. On the contrary we detected a slight decrease in LPS-induced apoptosis when adding L-NAME 5 mM, indicating a modest role of NO as a pro-apoptotic factor. The role of NO as a pro-apoptotic or an anti-apoptotic factor is controversial [26,27]; in fact, in several models including porcine endothelial cells [15], the administration of exogenous NO protected cells from apoptosis; nevertheless, the authors failed to obtain a protective effect by the inhibition of endogenous NO production. All these findings may suggest that the effect of NOS activity on apoptosis could involve several different pathways and only additional investigation will be able to assess whether the involvement of NO in our model was independent of the absolute amount of NO produced or was instead due to its cellular localisation.

## Competing interests

The authors declare that they have no competing interests.

## Authors' contribution

CB designed and performed research, analyzed the data and wrote the manuscript. FG performed research. AZ performed research, analyzed the data. MLB designed research, provided funding and edited the manuscript. ES partecipated in experimental design and provided funding. MF designed the experiment, analyzed the data, edited and approved the manuscript. All authors read and approved the final manuscript.
